# Tuberculosis in India

**Published:** 1909-05-22

**Authors:** 


The Hospital

A Journal of

The Medical Sciences and Hospital Administration.

New Series. No. 116, Vol. V. [No. 1188, Vol. XLVI.] SATURDAY, MAY 22, 1909.

### TUBERCULOSIS IN INDIA.

The *Indian Medical Gazette* for April contains a number of communications dealing with tuberculosis in India, being papers read before the Medical Section of the Asiatic Society. These papers remind us that India, in addition to being well supplied with her own particular tropical plagues, shares more amply even than ourselves in the visitations of the tubercle bacillus. The contributors all unite in deploring the inaccuracy of the statistics upon which they are forced to found their arguments; but with all allowances, it is clear that tuberculosis is very rampant in India. For example, 4 per cent. of all cases admitted to the General Hospital in Calcutta are admitted for tuberculosis. Again, the post-mortem records of the Medical College, Calcutta, go to show that evidence of tuberculosis was forthcoming in 25 per cent. of all bodies examined, while this disease was the actual cause of death in 17 per cent. Another writer states that the mortality from tuberculosis in Calcutta ranges from 2 to 3 per 1,000, while in Bombay City it reaches nearly 4 per 1,000. Comparison of this with our own figures—namely, 1.9 per 1,000 for England and Wales in the years 1901 to 1905—will bring home the horrible ravages of the disease in India.

Knowing what we do of the conditions which favour the spread of tuberculosis in communities, it is easy to believe that India offers many advantages to the tubercle bacillus. The population is intensely ignorant of hygiene, lives poorly, and exhibits (perhaps in consequence) a very limited resistance to pathogenic germs of various kinds. Moreover, overcrowding is even more habitual there than it is in our own slums, while the religious customs of a large section of the people involves a more or less close confinement of its female members. How efficient is this last item in promoting tuberculosis is well brought out by figures given by Mr. T. F. Pearse touching the ratio of mortality among men and women respectively. In Calcutta the mortality from tuberculosis is about 2 per 1,000 for males, but for females 3½ per 1,000. Further, Mahomedan women suffer much more than their Hindu sisters, the death-rate among Mahomedan women reaching nearly 6 per thousand. Nor is this high mortality confined to town dwellers. Mr. Chatterjee, assistant bacteriologist to the Medical College, Calcutta, asserts that it is as high in villages as elsewhere, and attributes the prevalence of the disease to the way the lower classes live, "huddled together in small huts around which dense vegetations are grown to effectually exclude light and air." An interesting point brought forward by this writer (and others) is the relative infrequency in India of those forms of surgical tuberculosis which are so common with us. This he attributes to the universal custom of boiling milk before using it as food. "So far as my knowledge goes," he says, "I have never seen anyone among our countrymen who uses unboiled milk unless it be on the express advice of medical men." There appears to be a consensus of opinion that surgical tuberculosis (that is, disease of bone and lymphatic glands in particular) is undoubtedly less common in India than it is with us. If the additional observation touching the universal custom of boiling milk is found to be justified, the conjunction will afford strong support to the often-suggested view that the surgical forms of tuberculosis depend in the main upon infection with the bacillus of bovine tuberculosis. In any case the evidence is good enough to stimulate precautions against the access of bovine bacilli to the human body.

The general conclusions drawn from the various papers read before the Section were that tuberculosis was a far greater scourge to Bengal than any purely tropical disease, and that there was urgent need for the provision of sanatoria for early cases. This, of course, is true enough, but it surely represents a very low ideal. It may be that ignorance of local conditions prompts the indulgence of impracticable aspirations, yet we should have been glad to see some expression of the patent fact that the extirpation of tuberculosis is not to be attained by the patching of invalids, but by the diffusion of knowledge concerning the means by which the disease is spread. Perhaps the speakers felt that under existing circumstances they were justified in merely bewailing the ignorance of the people, but to an outsider it appears a somewhat feeble proceeding. Now that the numbers of Indians who are not only educated, but medically educated, is becoming so considerable, it ought not to be impossible for them to institute a campaign for the teaching of their compatriots, particularly in the large towns. No one disguises the difficulty of the business, least of all those of us who have done our best to instil hygienic notions and the main data touching tuberculosis among our own poor. Even with our own relatively educated masses, it is an exasperating and discouraging task to attempt to urge the virtues of ventilation and cleanliness with regard to spitting. With such a population as that of India, the difficulties and discouragements must inevitably be multiplied many fold. Nevertheless, the doing of the thing is of prime importance, though progress be never so slow. We have heard much of late of the intellectual advances of the natives of India, and are quite content to credit their claims. The leaders of the community, both medical and lay, cannot find a better way of justifying their title to look after themselves than by combining to make a serious assault upon that ignorance which is the greatest bulwark of tuberculosis. The assault, if made at all, must be made, one imagines, by those who combine a self-sacrificing devotion to the cause with an intimate knowledge of the character and customs, fads and fetishes, of the uninstructed multitude. If this be true, the task must fall upon the shoulders of educated natives rather than upon the English, at least in so far as the actual proselytising of the people is concerned. It should not be impossible, though here again we speak with diffidence, to establish something after the fashion of Lady Aberdeen's travelling lecturers, who did such good work in Ireland, and we commend the suggestion to those who have local knowledge. Sanatoria are blessings which we are far from under-rating, but they barely touch the root of the evil. The natives of India can learn thus much from the experience of these islands.

